# Expression profiling of *Trypanosoma congolense* genes during development in the tsetse fly vector *Glossina morsitans morsitans*

**DOI:** 10.1186/s13071-018-2964-8

**Published:** 2018-07-03

**Authors:** Erick O. Awuoche, Brian L. Weiss, Paul O. Mireji, Aurélien Vigneron, Benson Nyambega, Grace Murilla, Serap Aksoy

**Affiliations:** 1 Department of Biochemistry, Biotechnology Research Institute, Kenya Agricultural and Livestock Research Organization, Kikuyu, Kenya; 2 Department of Biomedical Science and Technology, School of Public Health and Community Development, Maseno University, Private Bag, Maseno, Kenya; 3 Department of Epidemiology of Microbial Diseases, Yale School of Public Health, New Haven, CT USA; 4 Department of Agriculture, School of Agriculture and Food Science, Meru University of Science and Technology, Meru, Kenya; 5 Centre for Geographic Medicine Research – Coast, Kenya Medical Research Institute, Kilifi, Kenya; 6 Department of Medical Biochemistry, School of Medicine, Maseno University, Private Bag, Maseno, Kenya

**Keywords:** *Trypanosoma congolense*, *Glossina morsitans morsitans*, Gene expression analysis, Tsetse cardia, Tsetse proboscis and confocal microscopy

## Abstract

**Background:**

The tsetse transmitted parasitic flagellate *Trypanosoma congolense* causes animal African trypanosomosis (AAT) across sub-Saharan Africa. AAT negatively impacts agricultural, economic, nutritional and subsequently, health status of the affected populace. The molecular mechanisms that underlie *T. congolense*’s developmental program within tsetse are largely unknown due to considerable challenges with obtaining sufficient parasite cells to perform molecular studies.

**Methods:**

In this study, we used RNA-seq to profile *T. congolense* gene expression during development in two distinct tsetse tissues, the cardia and proboscis. Indirect immunofluorescent antibody test (IFA) and confocal laser scanning microscope was used to localize the expression of a putative protein encoded by the hypothetical protein (TcIL3000_0_02370).

**Results:**

Consistent with current knowledge, genes coding several variant surface glycoproteins (including metacyclic specific VSGs), and the surface coat protein, *congolense* epimastigote specific protein, were upregulated in parasites in the proboscis (PB-parasites). Additionally, our results indicate that parasites in tsetse’s cardia (C-parasites) and PB employ oxidative phosphorylation and amino acid metabolism for energy. Several genes upregulated in C-parasites encoded receptor-type adenylate cyclases, surface carboxylate transporter family proteins (or PADs), transport proteins, RNA-binding proteins and procyclin isoforms. Gene ontology analysis of products of genes upregulated in C-parasites showed enrichment of terms broadly associated with nucleotides, microtubules, cell membrane and its components, cell signaling, quorum sensing and several transport activities, suggesting that the parasites colonizing the cardia may monitor their environment and regulate their density and movement in this tissue. Additionally, cell surface protein (CSP) encoding genes associated with the Fam50 ‘GARP’, ‘iii’ and ‘i’ subfamilies were also significantly upregulated in C-parasites, suggesting that they are important for the long non-dividing trypomastigotes to colonize tsetse’s cardia. The putative products of genes that were upregulated in PB-parasites were linked to nucleosomes, cytoplasm and membrane-bound organelles, which suggest that parasites in this niche undergo cell division in line with prior findings. Most of the CSPs upregulated in PB-parasites were hypothetical, thus requiring further functional characterization. Expression of one such hypothetical protein (TcIL3000_0_02370) was analyzed using immunofluorescence and confocal laser scanning microscopy, which together revealed preferential expression of this protein on the entire surface coat of *T. congolense* parasite stages that colonize *G. m. morsitans*’ proboscis.

**Conclusion:**

Collectively, our results provide insight into *T. congolense* gene expression profiles in distinct niches within the tsetse vector. Our results show that the hypothetical protein TcIL3000_0_02370, is expressed on the entire surface of the trypanosomes inhabiting tsetse’s proboscis. We discuss our results in terms of their relevance to disease transmission processes.

**Electronic supplementary material:**

The online version of this article (10.1186/s13071-018-2964-8) contains supplementary material, which is available to authorized users.

## Background

Tsetse (*Glossina* spp.)-transmitted *Trypanosoma congolense* is a major cause of animal African trypanosomosis (AAT) in livestock in most of sub-Saharan Africa. The disease has a significant economic and public health impact due to its wide geographical distribution and broad vertebrate animal host range [1, 2]. To date no vaccines against *T. congolense* exist. Thus, AAT management relies on controlling the tsetse vector *via* use of baited traps and targets, spraying with insecticides and/or treatment of infected animals using trypanocidal chemotherapy. Inherent limitations underlying successful application of these strategies include drug resistance in the parasite [3–7] and re-infestation by residual tsetse populations where and when control programs are abandoned [8]. These limitations necessitate development of novel intervention strategies that may interfere with parasite transmission through the tsetse vector. Accordingly, additional information on genetic factors that regulate *T. congolense* development in the tsetse fly is required.

African trypanosomes (*T. congolense*, *T. vivax* and *T. brucei*) must infect vertebrate hosts and tsetse vectors in order to complete their life-cycle. To do so the parasites must progress through a series of developmental forms adapted to each life-cycle stage. Vertebrate infectious metacyclic *T. congolense* and *T. vivax* develop in tsetse’s proboscis (PB) while metacyclic *T. brucei* form in the fly’s salivary glands (SG). In the mammalian host, *T. congolense* proliferates as single type of bloodstream form (BSF) parasite covered with carbohydrate-rich variant surface glycoproteins (VSGs) [9]. Upon entry into the lumen of tsetse’s gut, BSF *T. congolense* differentiate to procyclic forms (PCF), and this transformation is accompanied by changes in parasite morphology, a switch from glycolysis to oxidative phosphorylation for energy metabolism and replacement of the VSG surface coat with one composed of procyclins [10]. In successful infections, the parasites migrate to tsetse’s cardia, transform into long non-dividing trypomastigote parasites and subsequently migrate to and colonize the fly’s PB. Finally, these cells differentiate into epimastigote forms (EMF), which attach to the PB wall and give rise to mammalian infective metacyclic forms (MCFs) [11, 12]. Molecular factors that regulate differentiation of *T. congolense*, and major barriers to *T. congolense* transmission through the tsetse vector, are poorly understood. Sequencing and annotation of the genomes of the three African trypanosomes and six species of tsetse fly, along with advances in RNA sequencing (RNA-seq) methodologies, have significantly expanded our knowledge of the molecular biology that underlies this vector-pathogen system. However, most research to date has been performed using *T. brucei* [13–22], with two RNA-seq and one proteomic study reported in *T. vivax* [23, 24] and a single transcriptomic and proteomic analysis performed with in vitro cultured *T. congolense* cells [25, 26].

In this study, we utilized high throughput RNA-seq to profile expression of *T. congolense* genes in infected cardia and PB organs of *G. morsitans morsitans*. We mined the data to identify molecular factors that may be functionally involved in vector-parasite interactions, parasite differentiation and maintenance of *T. congolense* in tsetse’s cardia and PB. We established that 15.95% of all genes were expressed at significantly different levels in C-parasites compared to PB-parasites. More specifically, genes upregulated in C-parasites encoded transporter and Fam50 (‘GARP’, ‘iii’ and ‘i’ subfamily) proteins, while those upregulated in PB-parasites encoded VSGs among other proteins associated with parasite multiplication. We discuss our findings in the context of furthering new initiatives for development of novel disease control applications.

## Methods

### Trypanosome strain, tsetse flies and tsetse infection

*Trypanosoma congolense* [Trans Mara strain, variant antigenic type (VAT) TC13] used in this study were kindly provided by Professor Utpal Pal, at Department of Veterinary Medicine, University of Maryland. The parasite was originally isolated from an infected bovine in Trans Mara, Kenya [27]. The VAT TC13 was obtained by serial cloning of the parasite in immunosuppressed CD1 mice [28]. The BSF TC13 was amplified in rats and harvested from blood at peak parasitemia by cardiac puncture.

Tsetse flies (*Glossina morsitans morsitans*) used in this study were reared in Yale University (New Haven, CT, USA) insectary at 24 °C and 50% relative humidity. In this colony, tsetse flies are fed at 48 hour intervals using an artificial membrane-based feeding system [29] with defibrinated bovine blood commercially supplied by Hemostat Laboratories (Dixon, CA, USA). Teneral (newly eclosed and unfed) *G. m. morsitans* males were infected by feeding them a blood meal supplemented with BSF *T. congolense* (VAT TC13) (8 × 10^6^ parasites per ml of blood *via* artificial membrane feeding method [29]). Subsequently, the flies were maintained on normal blood meal for 28 days until tissue dissection was performed.

### Dissection and collection of tsetse tissues

The cardia and probosces of *T. congolense* infected flies were dissected 28 days post-challenge and 72 h after the last blood meal. Dissections of cardia for *T. congolense* infection analyses were microscopically conducted in PSG buffer (pH 8.0) using Zeiss Axiostar Plus light microscope (Carl Zeiss Light Microscopy, Gottingen) at ×400 magnification. Two biological replicates of infected probosces (consisting of 130 probosces each) from a recent study were used [30]. Infected cardia were immediately placed in TRIzol (Thermo Fisher Scientific Inc., CA, USA). Three biological replicates consisting of 15 infected cardia each were used. Only flies which had both cardia and proboscis infections were used for all downstream analyses.

### RNA extraction, cDNA library preparation and sequencing

Total RNA extraction and subsequent elimination of contaminating DNA was done using TRIzol and Turbo-DNase (Thermo Fisher Scientific Inc., CA, USA), respectively, following the manufacturer’s protocol. Elimination of DNA from the total RNA was confirmed by PCR using both *T. congolense-* and *G. m. morsitans-*specific *beta*-tubulin and glyceraldehyde-3-phosphate dehydrogenase (*gapdh*) primers for trypanosome and tsetse fly, respectively. RNA quantity and quality were determined using a Bioanalyzer 2100 (Agilent, Palo Alto, CA, USA) and subsequent cDNA libraries prepared using a NEBNext Ultra Directional RNA Library Prep Kit (New England Biolabs, Inc., MA, USA) according to the manufacturer’s protocol. The cardia and probosces libraries were barcoded for Illumina HiSeq 2000 sequencing (Illumina, Inc., CA, USA) (unpaired 75 bases) at Yale Center for Genome Analysis (YCGA, New Haven, CT). For the generation of these cDNA libraries, 900 ng of high quality (RNA integrity number > 7.0) total RNA samples was used. The Sequence Read Archive number at NCBI of infected cardia is SRP093558 and that of infected probosces is PRJNA354110 [30].

### Processing and differential analyses of *T. congolense* transcriptome

All bioinformatics analyses were conducted using CLC Genomics Workbench version 8.5 (CLC bio, Cambridge, MA). The fasta RNA-seq data were first processed for quality conformity and then mapped to *T. congolense* IL 3000 reference strain transcripts version 9 [31] obtained from TritrypDB [32]. The TC13 strain used in this study was different from the strain from which the whole genome data were generated, but both parasite strains had originated from Trans Mara in Kenya [33]. The mapping employed algorithms that allowed for only two mismatches and a maximum of 10 hits per read with at least 80% of the reads matching the gene at 95%. Reads per kilobase per million mapped (RPKM) [34] was used as a proxy of gene expression. Differentially expressed (DE) genes were determined using RNA-Seq module employing Baggeley’s test and Bonferroni analysis [35]. Relative fold change (FC) between parasite genes from the cardia and proboscis was calculated as a ratio of their respective RPKM values. Differential expression analyses were also conducted using EdgeR software [36] to corroborate the results obtained by CLC Genomics Workbench. A conservative selection regime was adopted to minimize false detections of differential expression. Within this regime, transcripts were considered DE between parasites in cardia and probosces if they had (i) at least two-FC in either of the tools used (CLC-genomics or EdgeR), (ii) normalized false detection rate (FDR) corrected *P*-value of ≤ 0.05, 3) supported by at least 30 reads mapping in either library (C-parasite or PB-parasite), (iv) at least five RPKM, and (v) considered as DE with both tools (CLC-genomics and EdgeR).

### Functional annotation of DE genes

Functional annotations of DE genes were conducted using Blast2GO version 3.4 program [37–39]. Briefly, homology searches for all *T. congolense* genes were conducted against NCBI non-redundant (nr) protein database using BLASTx [40] with an E-value BLAST cut-off of 1.0E-03. The blast results were then mapped and annotated for gene ontology (GO) categories related to biological processes, molecular function and cell component using an algorithm in Blast2GO software. Enriched GO terms among DE gene products were established by Fisher’s exact test at a false discovery rate (FDR) *P*-value ≤ 0.05 [38]. Protein domains/signatures associated with putative products of the DE genes were determined through InterProScan analysis [41]. The DE genes encoding proteins with predicted cell-surface functions were retrieved from previously published data [42] and validated using PredGIP [43], FragAnchor [44] and BigPI [45] softwares for GPI-anchor motifs prediction, and TMHMM [46] software for *trans*-membrane helices prediction. Secreted proteins were predicted using SignalP version 1.4 software [47]. Enriched metabolic pathways for gene products were identified using KEGG [48, 49] and TrypanoCyc [50] tools implemented in TritrypDB at FDR ≤ 0.05.

### Real time quantitative-PCR (RT-qPCR) analysis for transcriptome validation

RT-qPCR analysis was conducted on independent samples to corroborate RNA-seq data. Samples for transcriptome validation were obtained from an independent set of *T. congolense* infected tsetse cardia or probosces. Total RNA and DNase treatment of samples of infected cardia or probosces were prepared as described above. Eight biological replicates of infected cardia or probosces were used, each containing a pool of 5 and 25 tissues, respectively. cDNA was synthesized with oligo-dT primers and random hexamers using iScript cDNA synthesis reaction kit (Bio-Rad, CA) according to the manufacturer’s protocol. RT-qPCR was performed in technical duplicate (for each biological replicate) on eight selected DE genes (Additional file 1: Text S1). All RT-qPCR results were normalized using *T. congolense gapdh* (TcIL3000_10_5910) and 60S ribosomal (TcIL3000_0_32580) genes determined from each biological replicate. The two genes were more stable in these two tissues with standard deviation of the crossing point being less than 1 according to BestKeeper analysis [51]. Correlation between RT-qPCR and RNA-seq fold change results were evaluated using Pearson correlation analysis (Additional file 2: Text S2).

### Semi-quantitative RT-PCR analysis of gene expression

Semi-quantitative RT-PCR was performed for selected cell surface protein (CSP) encoding genes using cDNA samples synthesized from RNA isolated from parasites obtained from infected rats, tsetse midgut, cardia or probosces, following a previously published method [52]. To normalize cDNA samples from various tsetse tissues and BSF for PCR analysis, equal amounts of samples were diluted 10-fold and run for 28 PCR cycles using *T. congolense* specific *gapdh* primers. The PCR normalization of samples using *gapdh* was performed at the following cycling conditions: 2 min at 95 °C followed by 28 cycles at 95 °C for 45 s, 55 °C for 1 min and 72 °C for 1 min. A final 75 °C extension was performed for 10 min. For experimental analysis, all PCR reactions were performed as technical replicates as described at 34 cycles for each sample (from infected rats, tsetse midgut, cardia or probosces cDNAs) and the PCR products individually resolved on 1% agarose gel. As control for experimental analysis, *gapdh* gene was also amplified at 34 PCR cycles. Gel visualization, image capturing and semi-quantitative measurement of expression variation between different tissue samples was done using Gel Doc™ XR+ Gel Documentation System (Bio-Rad, CA, USA). The expression values of each gene for each parasite stage were normalized to those of similarly treated *gapdh* controls. Fold change for each of the three-developmental tsetse tissue-specific and BSF samples was calculated as described by Savage et al. [52]. Primers for each gene used in this analysis can be found in Additional file 1: Text S1.

### Recombinant protein expression, purification and antibody production

The gene encoding a hypothetical Fam50 protein, (TcIL3000_0_02370), was cloned into pET28a expression vector without signal peptide and GPI-anchor domains, sequenced, and expressed in BL21 competent bacterial cells (Promega, Madison, WI). For cloning, *BamHI* and *XhoI* enzyme restriction site sequences were added to the forward and reverse primers respectively (Additional file 1: Text S1). For recombinant expression, the putative 224 amino acids (26 to 249 from the N-terminal) was cloned. Recombinant protein expression was induced with 1 mM isopropyl-β-thiogalactoside at 28 °C, purified using 6× His-tag pull down and analyzed by polyacrylamide gel electrophoresis for purity. The rec-protein was then concentrated by centrifugation in a PM-membrane of 10 MW cut-off (Thermo Fisher Scientific Inc., CA, USA). Recombinant protein yield was determined by BCA kit (PIERCE Chemical Company, Rockford, IL, USA). Polyclonal rabbit antibodies against recombinant TcIL3000_0_02370 protein was commercially generated by Cocalico Biologicals, Inc., PA, USA.

### Immunofluorescence assay (IFA) and microscopic observation

IFA was performed to localize expression of TcIL3000_0_02370 CSP. Tsetse-derived *T. congolense* parasites harvested from infected tsetse midgut, cardia or proboscis organs 28-days post-infections were individually placed in PSG buffer for 5–10 min to enable parasites to diffuse out of the tissues and into the buffer. Parasites in the buffer were then spotted onto a glass slide and cells fixed with 4% paraformaldehyde at room temperature for 30 min. To permeabilize the samples, slides were placed 10 min in 0.1% Triton X-100 PBS and then washed twice in PBS prior to the blocking step. Both permeabilized and non-permeabilized samples were blocked with 5% bovine serum albumin (BAS) in PBS for 30 min and then incubated overnight at 4 °C with rabbit anti-TcIL3000_0_02370 polyclonal antibody diluted 1:100 in 5% BSA in PBS. Slides were then washed twice for 5 min in 5% BSA in PBS at RT prior to being incubated for 1 h at RT with goat anti-rabbit Alexa596-conjugated secondary antibodies diluted 1:500 in 5% BSA in PBS. Slides were then washed twice for 5 min in PBS at RT before being quickly washed in ultrapure water to remove excess salts. Slides were air-dried, mounted with VectaShield H-1500, stained with 1 μg/ml DAPI (to visualize the cells’ nucleus and kinetoplast) and washed twice with PBS (pH 8.0) as described above. Slides were observed with a Zeiss LSM 710 confocal microscope (Zeiss, Germany). Images were merged and contrasts-treated using the module Fiji for the ImageJ software [53].

## Results

### Abundance of *T. congolense* transcripts in infected cardia and proboscis

We performed RNA-seq analyses of *T. congolense* in infected tsetse cardia (C-parasites) and proboscis (PB-parasites) organs and characterized the expression of parasite specific transcripts. Results from the infected tissues revealed that 7.34% and 4.04% of the total reads obtained from the infected cardia and proboscis transcriptomes, respectively, mapped to the putative *T. congolense* IL3000 gene sets. These included genes encoding putative *T. congolense* VSGs (Additional file 3: Figure S1a). Upon mapping, transcripts were detected for 91.8% of the 13,549 *T. congolense* IL3000 genes. Most of these genes (74% and 66% of the C- and PB-parasites, respectively) exhibited low transcript abundance (≤ 100 total reads). An average of 1.12% of the genes were categorized as having high transcript abundance (>10,000 total reads) in both libraries (Additional file 3: Figure S1b).

We next examined the genes comprising the top 99 percentile (RPKM) in each tissue (C-parasite or PB-parasite) stage to identify the most abundantly expressed genes in each tissue, and assess the extent of overlap. This analysis identified 131 genes, of which 56 were common between C- and PB-parasites. The remaining 75 genes were in the top RPKM percentile in one of the tissue-specific parasites (Additional file 3: Figure S1c, Additional file 4: Table S1). Gene ontology enrichment analysis of the putative shared gene products showed enriched terms broadly related to ribosome, translation, transport and GTPase activity (Table 1), indicating abundant protein synthesis and transport activity. Metabolic pathway analysis of the shared abundant products identified cysteine and methionine, aspartate superpathway, methionine salvage cycle, glycerol degradation, trypanothine and S-adenosylmethionine biosynthesis, as well as peroxide metabolic pathways, as enriched (Table 1). The methionine cycle recycles methionine from methylthioadenosine, which is a by-product of polyamine synthesis. The activity of this cycle in recycling methionine was previously proposed in *T. brucei* [54]. However, the methionine cycle is now thought to be inactive in trypanosomes, at least in PCF and BSF *T. brucei* parasite stages [55, 56]. The functionality of this pathway remains to be elucidated in other trypanosome stages and species, including *T. congolense*. Degradation of glycerol, a carbon source likely present in insect vectors, and enrichment of the aspartate superhighway, could mean that these two pathways may serve as the source of acetate in lipid biosynthesis. High expression of putative proteins functionally linked with trypanothione and peroxide metabolic pathways may be important for the parasites to survive in conditions under high oxidative stress [57–59] that may exist in infected cardia and probosces.

**Table 1:** Gene Ontology and metabolic enriched pathways of the 99-percentile common most highly expressed gene products

Gene ontology enriched pathway						
Category	GO ID	Term	FDR	*P*-value	Test^a^	Ref^b^
Cell component	GO:0005840	Ribosome	7.98E-09	3.56E-12	15	172
	GO:0022627	Cytosolic small ribosomal subunit	1.77E-04	5.93E-07	3	3
	GO:0000314	Organellar small ribosomal subunit	4.84E-02	7.12E-04	2	5
Molecular function	GO:0003735	Structural constituent of ribosome	1.89E-08	1.26E-11	14	154
	GO:0015114	Phosphate ion transmembrane transporter activity	9.55E-03	7.24E-05	2	2
	GO:0005315	Inorganic phosphate transmembrane transporter activity	9.55E-03	7.24E-05	2	2
	GO:0003924	GTPase activity	1.35E-02	1.11E-04	5	57
	GO:0004478	Methionine adenosyltransferase activity	2.11E-02	2.16E-04	2	3
	GO:0019843	rRNA binding	2.87E-02	3.07E-04	3	16
	GO:0005515	Protein binding	2.92E-02	3.19E-04	10	325
	GO:0005200	Structural constituent of cytoskeleton	3.33E-02	3.71E-04	3	17
	GO:0015224	Biopterin transporter activity	3.33E-02	4.30E-04	2	4
Biological processes	GO:0006412	Translation	9.19E-07	1.43E-09	15	261
	GO:0006817	Phosphate ion transport	9.55E-03	7.24E-05	2	2
	GO:0000028	Ribosomal small subunit assembly	9.55E-03	7.24E-05	2	2
	GO:0006915	Apoptotic process	1.67E-02	1.60E-04	3	13
	GO:0006556	S-adenosylmethionine biosynthetic process	2.11E-02	2.16E-04	2	3
	GO:0015877	Biopterin transport	3.33E-02	4.30E-04	2	4
	GO:0006986	Response to unfolded protein	3.33E-02	4.30E-04	2	4
	GO:0051258	Protein polymerization	4.35E-02	6.11E-04	3	20
Metabolic pathways						
Database	Pathway ID	Pathway description	FDR	P-value	Test^a^	Ref^b^
KEGG	ec00270	Cysteine and methionine metabolism	1.64E-02	1.64E-02	2	74
TrypanoCyc	SAM-PWY	S-adenosyl-L-methionine biosynthesis	3.74E-04	6.24E-05	2	3
	PWY-802	methionine and S-adenosylmethionine synthesis	3.74E-04	9.35E-05	2	4
	PWY0-781	aspartate superpathway	5.96E-04	2.24E-04	2	7
	PWY1V8-2	Methionine salvage cycle	8.17E-04	4.09E-04	2	10
	PWY1V8-6	Trypanothine biosynthesis and peroxide metabolism	1.04E-03	6.48E-04	2	13
	METHIONINE-DEG1-PWY	methionine degradation I (to homocysteine)	1.05E-02	7.84E-03	2	50
	PWY0-381-1	glycerol degradation I	1.27E-02	1.11E-02	1	3

^a^Genes in the top 99-percentile RPKM dataset

^b^Entire trypanosome genes in TritrypDB database in respective metabolic pathway

Of the remaining 75 most abundant transcripts, 33 were present only in the top 1% RPKM of the C-parasite library. These putatively encoded Glycosomal phosphoenolpyruvate carboxykinase, Zinc finger protein (ZC3H36), Cytochrome oxidase IV, Cysteine peptidase, and several transporter and receptor-type adenylate cyclase proteins. In addition, putative cell surface proteins, such as Procyclin-like and GARP proteins, were most abundant in C-parasites (Additional file 4: Table S1). The remaining genes (42 out of 75) were most abundantly expressed in PB-parasites and they included transcripts encoding 23 ribosomal proteins, three histones (H2A, two-H2B), two actins, and several cell surface proteins (Additional file 4: Table S1). Thus, the PB-parasite data imply an increased rate of protein synthesis at this stage, which is an important phenomenon during active cell division.

### Differential expression of *T. congolense* genes and enrichment analysis

**Fig. 1:**
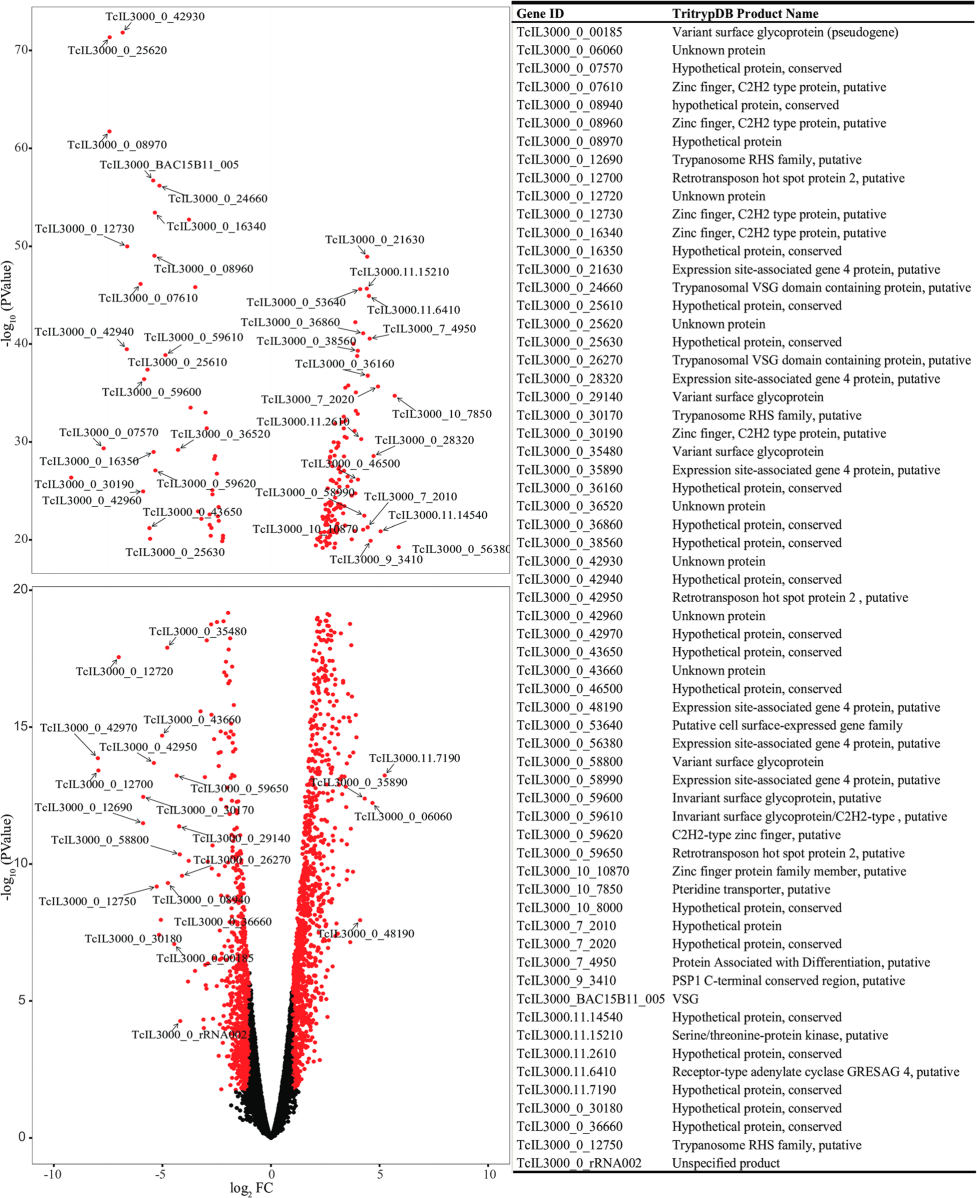
A Volcano plot showing differentially expressed genes of *T. congolense* parasites in the cardia relative to those in the probosces of *G. morsitans.* Only transcripts with at least 30 reads mapped and at least five RPKM (Reads Per Kilobase Million) by CLC-Genomics and EdgeR analysis [36, 99] in either library (C-parasites or PB-parasites) were considered. Red dots indicate DE genes with an FC of ≥ 2 (log_2_ = 1) and false detection rate (FDR) corrected *P-*value of < 0.05 between cardia and proboscis parasites. The x-axis displays magnitude of fold-changes and y-axis the statistical significance (-log_10_ of *P-*value). Points having FC of < 2 (log_2_ < 1) on an FDR corrected *P-*value of < 0.05 are shown in black, and indicate genes with non-significance change between different developmental states

To characterize potential molecular differences between trypanosome developmental stages in cardia and proboscis, we analyzed DE genes between C- and PB-parasite libraries. This comparison identified 2131 (15.95%) DE genes, of which 59.17% exhibited significantly higher expression in C-parasites relative to PB-parasites (Fig. 1, Additional file 5: Table S2). Because of the limited number of biological replicates used, the DE genes that exhibit low FC (i.e. ≤ 2) requires verification. The DE genes with the greatest fold change in favor of C-parasites encoded receptor-type adenylate cyclases, transporter proteins (pteridine, amino acid and ABCs), putative cell-surface protein, procyclin-like protein, protein associated with differentiation 5 (PAD5) and zinc finger ZC3H22 protein. Similarly, VSG, two invariant surface glycoproteins, three histones (two-H2B and H4), zinc finger type C2H2 proteins and a number of hypothetical proteins were among those with the greatest fold change in PB- compared to C-parasites (Fig. 1, Additional file 5: Table S2). RT-qPCR validation of our RNA-seq data revealed significant correlation (Pearson correlation = 0.9915) between these two analytical methodologies (Additional file 2: Text S2).

Blast2GO Fishers exact test of DE genes revealed 37 and five enriched Gene Ontology (GO) terms associated with C- and PB-parasites, respectively (Additional file 6: Table S3). The GO enriched terms of putative proteins upregulated in C-parasites were broadly associated with nucleotides (nucleotide binding and cyclic nucleotide biosynthetic process; the energy carriers), ATP binding, protein phosphorylation, signaling, cell (integral component of cell membrane, membrane region, cytoplasmic side of plasma membrane), adenylate cyclase activity, quorum sensing as well as microtubules, protein serine/threonine and transport processes. This suggests that C-parasites are energetically active and can monitor and respond to their surroundings. Induction of several transport pathways in C-parasites suggests that these trypanosomes may be scavenging for nutrients in the cardia. The putative products of genes upregulated in the PB-parasite were associated with nucleosome, cytoplasmic part, membrane bound organelle, protein folding and protein heterodimerization activity; terms possibly reflecting high rate of cell division by parasites residing in this organ.

### Metabolic pathways

**Fig. 2:**
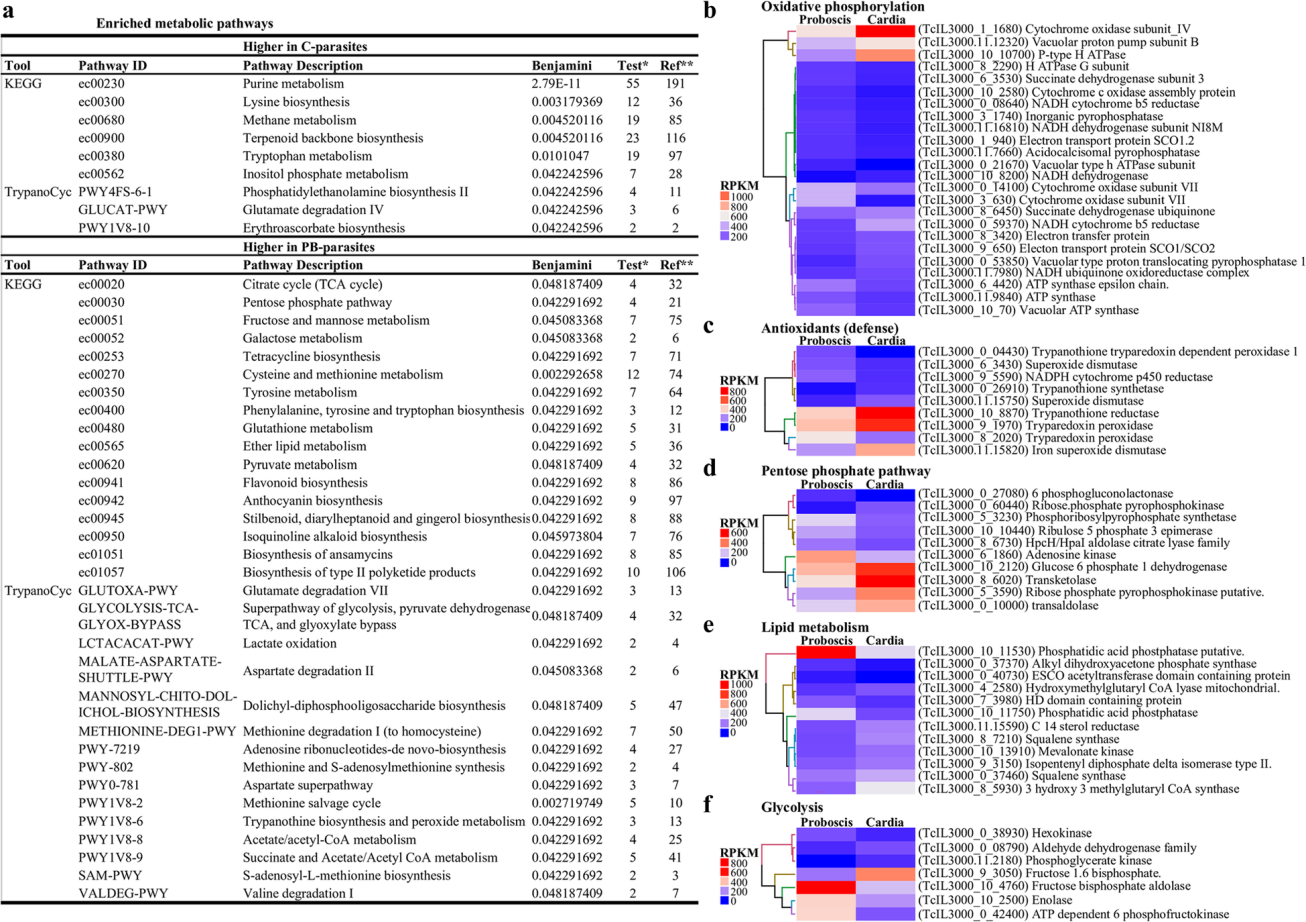
Differential expression of genes in metabolic pathways. Heat maps showing the expression profiles consisting of respective RPKMs clustered using Euclidean distance calculation and ward.D clustering methods. **a** KEGG and TrypanoCYC enriched metabolic pathway. **b**-**f** Genes that function in the oxidative phosphorylation pathway, antioxidant (defense), pentose phosphate pathway, lipid metabolism, and glycolytic pathway respectively

African trypanosomes live in a glucose-rich environment in the mammalian hosts and in a glucose deficient environment in the tsetse vector. These contrasting habitats require trypanosomes to adjust their metabolic processes for energy production. Specifically, for energy production, *T. congolense* BSF parasites rely entirely on glycolysis and substrate level phosphorylation whereas midgut PCF parasites utilize oxidative phosphorylation. The KEGG [48] and TrypanoCyt [50] analyses of putative products of DE genes for enriched metabolic pathways identified pathways broadly linked with protein/amino acid metabolism (Fig. 2a). A survey of individual genes identified those that encoded proteins putatively associated with the Krebs cycle, respiratory chain and oxidative phosphorylation to be DE between C-parasites and PB-parasites (Fig. 2b), suggesting an enhanced respiratory activity of *T. congolense* stages in both tsetse tissues. Trypanosome respiratory activity, coupled with the reactive oxygen intermediates produced by trypanosome infected tsetse tissues [57, 58], can result in the production of reactive oxygen species that leads to oxidative stress to both tsetse fly and trypanosomes. As such, we investigated if parasites in these two tissues produce antioxidants and NADPH that may protect them from oxidative damage. In this regard, we identified putative proteins that likely function as antioxidants (Fig. 2c) and in the pentose phosphate pathway (Fig. 2d), of which some were upregulated in C-parasite and others in PB-parasites. Pentose phosphate pathway is an essential maintenance pathway that leads to generation of reducing agents in the form of NADPH (which act as an electron donor in detoxification reaction). The cells may use this pathway not only to protect themselves against oxidative stress, but also to produce ribose phosphates that function in nucleotide synthesis.

Putative proteins linked to lipid metabolism involving sterol biosynthesis and ether lipid metabolism were upregulated in C- and PB-parasites, respectively (Fig. 2e). Increased expression of sterol synthesis genes and desaturase by C-parasites may enable the generation of polyunsaturated fatty acids that may help to maintain parasite membrane fluidity under variable temperature conditions in the insect host as they move to the PB where they start to divide. Induced expression of lipid metabolism genes in PB-parasites suggest an increased activity of this pathway, probably indicating high lipid utilization for energy. Lastly, gene products that function in the glycolytic pathway were also DE (Fig. 2f). The majority of them, notably hexokinase, phosphofructokinase, fructose-bisphosphate aldolase and enolase, were upregulated in PB-parasites. The enhanced expression of hexokinase by the PB-parasites may be an indication of pre-adaptation of these parasites for the glucose-rich bloodstream environment in their mammalian host.

### Expression of genes regulating trypanosome differentiation

**Fig. 3:**
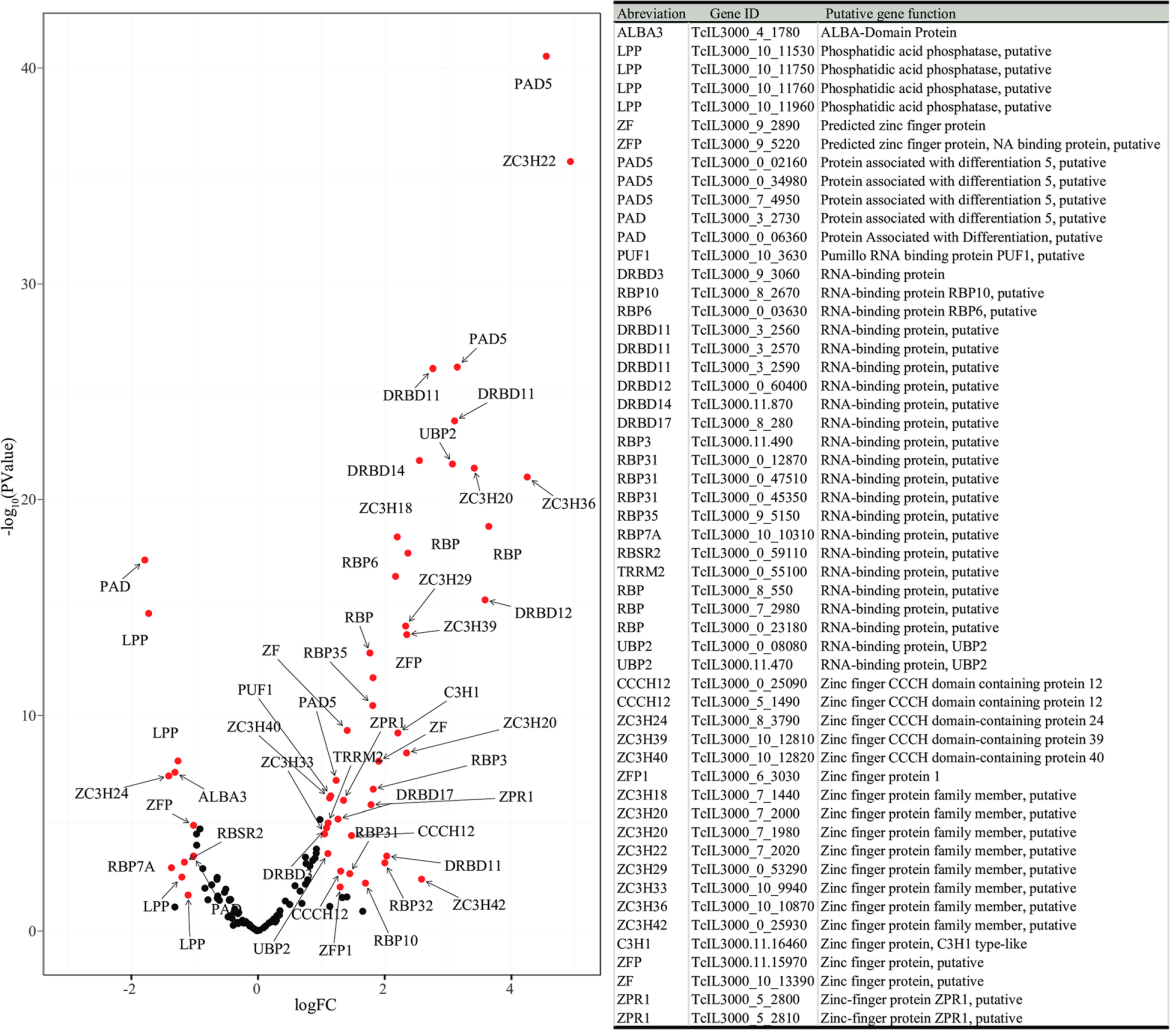
Volcano plot showing DE genes between cardia and proboscis parasites encoding proteins putatively associated with trypanosome differentiation. The red dots indicate points-of-interest with a fold-change of ≥ 2 (log_2_ = 1) and False Detection Rate (FDR) corrected *P* < 0.05 between cardia and proboscis parasites. Black dots represent genes with no significance change in expression level

*Trypanosoma congolense* differentiates into several forms during development in tsetse before becoming mammalian infective MCFs in the PB. In *T. brucei,* this process is regulated by a number of proteins, including RNA-binding proteins (RBPs) [59], lipid phosphate phosphatases (LPP) [60] and/or Proteins Associated with Differentiation (PADs), also known as Major Facilitator Superfamily (MFS) transporters which are expressed in the mammalian stage of trypanosomes [61]. We identified 44 RBPs, five PADs and four LPPs that were DE (Fig. 3, Additional file 7: Table S4). Most of these RBPs were highly expressed in the C-parasites relative to PB-parasites. Even though most of the RBPs, especially in *T. congolense*, are not functionally annotated, it is worth noting that ZC3H20 and RBP6 were abundant in C-parasites. In *T. brucei,* ZC3H20 is required for growth of PCF, while RBP6 is involved in differentiation to MCF [62, 63]. The RBPs that exhibited higher expression in PB-parasites relative to C-parasites included ALBA3, RBSR2,3, RBP7A and Zinc finger protein (ZC3H24). ALBA3 in *T. brucei* is expressed by all parasite stages except for those in the cardia, and artificial overexpression of this protein disturbs normal trypanosome development process taking place in the cardia [64]. All DE PADs were highly expressed in C-parasites compared to PB-parasites, with the exception of only two that were upregulated in PB-parasites. In contrast to RBPs and PADs, the expression of all LPPs was higher in PB-parasites relative to C-parasites, mirroring results observed in *T. brucei* in tsetse’s SG [60].

### Differential expression of *T. congolense* cell-surface proteins (CSPs)

**Fig. 4:**
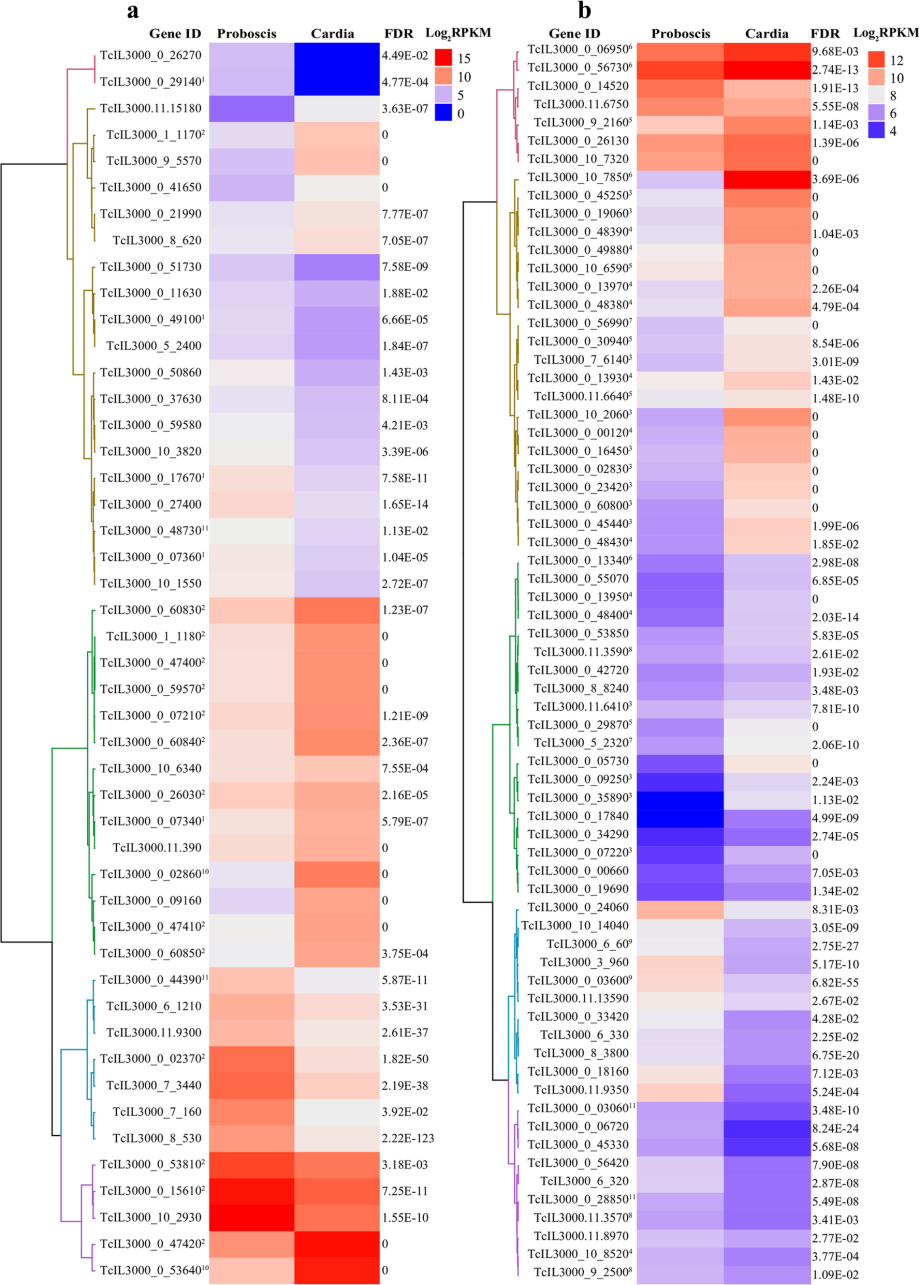
Heatmaps of expression of *T. congolense* genes encoding putative cell surface proteins. **a** GPI anchored proteins. Prediction of the putative GPI-anchored cell surface proteins was determined by FragAnchor [44], PredGPI [43] and BigPI [45]. **b** Transmembrane proteins. *Trans*-membrane helices was predicted using TMHMM [46]. The expression profiles consist of respective log_2_ transformed RPKM clustered using Euclidean distance calculation and ward.D clustering methods. ^1^Fam13/16 (VSGs), ^2^Fam50 (Brucei alanine-rich protein), ^3^Fam51 (Expression site-associated gene 4), ^4^Fam54 (Amino acid transporter), ^5^Fam56 (ABC transporter), ^6^Fam57 (Folate transporter, ESAG10), ^7^Fam60 (Membrane transporter protein), ^8^Fam61 (Nucleoside transporter), ^9^Fam67 (Cysteine peptidase), ^10^Fam12 (Procyclin-like), ^11^Fam14 (Transferrin receptor-like, PAG-like)

The surface of *T. congolense* is covered by a dense layer of glycoprotein, the composition of which is characteristic of each differentiation stage. Most of these glycoproteins are attached to the plasma membrane by a glycosylphosphatidyl inositol (GPI) anchor or are transmembrane (TM), and have been grouped into multigene families [42]. Of the DE gene dataset, 223 encoded CSPs based on the presence of putative GPI-anchor or TM protein domains (Fig. 4, Additional file 8: Table S5). For the putative GPI-anchored proteins (Fig. 4a), our analyses show that all the six genes putatively encoding ‘GARP’ and ‘iii’ subfamily proteins, and the single protein of ‘i’ subfamily of the Fam50 [42], were upregulated in C-parasites. In addition, two Fam12, four Fam47, three Fam51 and several hypothetical GPI-anchored protein coding genes were also induced in C-parasites. The GPI-anchored protein-encoding genes induced in the PB-parasites included ten putative VSG proteins, of which three were metacyclic-specific VSGs [m-VSG-3, 6, 10; (Fig. 4b)]. In the C-parasite dataset, one upregulated gene encoded a putative VSG (TcIL3000_0_07340). Apart from the VSGs, other upregulated genes in PB-parasites that encoded putative GPI-anchored CSPs included Fam50’s three ‘CESP’ subfamily genes, the single (TcIL3000_0_02370) subfamily ‘iv’ gene as well as two Fam46 (major surface protease gp63) and Fam14 (procyclin-associated gene; PAG1-2,4-5) [42]. Also, upregulated in PB-parasites were genes encoding a haptoglobin-hemoglobin receptor protein, an amastin surface glycoprotein and several hypothetical proteins.

For the genes encoding putative TM proteins, we found 14 members of Fam51, several members of transporter families (Fam54, 56, 57, 59, 60, 61, 62), a lipase (Fam63) and a zinc-finger (Fam76) to be highly expressed by the C-parasites compared to PB-parasites (Fig. 4b, Additional file 8: Table S5). On the other hand, in the PB-parasites, at least a member of different gene families was induced relative to the C-parasite (Fig. 4b, Additional file 8: Table S5). Of these gene families, Fam67 (cysteine proteases) attracted considerable interest [65], more so in *T. congolense* [66], where they are thought to have a direct impact on disease pathogenesis as virulence factors involved in host invasion, migration, metabolism and immune evasion. Expression of cysteine protease encoding genes by the insect stage parasites mirrors results found in previous proteome [26] and transcriptome [25] studies in the insect stages, suggesting that these proteins may also play important roles vector-parasite interactions. Importantly, genes highly expressed by PB-parasites are likely to encode proteins that are transmitted to mammalian hosts, and this may assist the parasite in overcoming the many defense factors produced by the host immediately upon their entry in the animal.

The *T. congolense* genome also encodes parasite species specific CSPs. These *T. congolense* unique CSPs (grouped into Fam17-22) may help distinguish this parasite from *T. brucei* and *T. vivax* [42]. Analysis of these genes revealed that they exhibited relatively low expression levels in both C- and PB-parasites (Additional file 9: Figure S2). Nonetheless, some members of Fam17 and Fam18 were upregulated in the C-parasites relative to PB-parasites, while three members of Fam20 and a member of Fam21 showed higher expression in PB-parasites relative to C-parasites.

### Developmental stage-regulated expression of selected CSPs

**Fig. 5:**
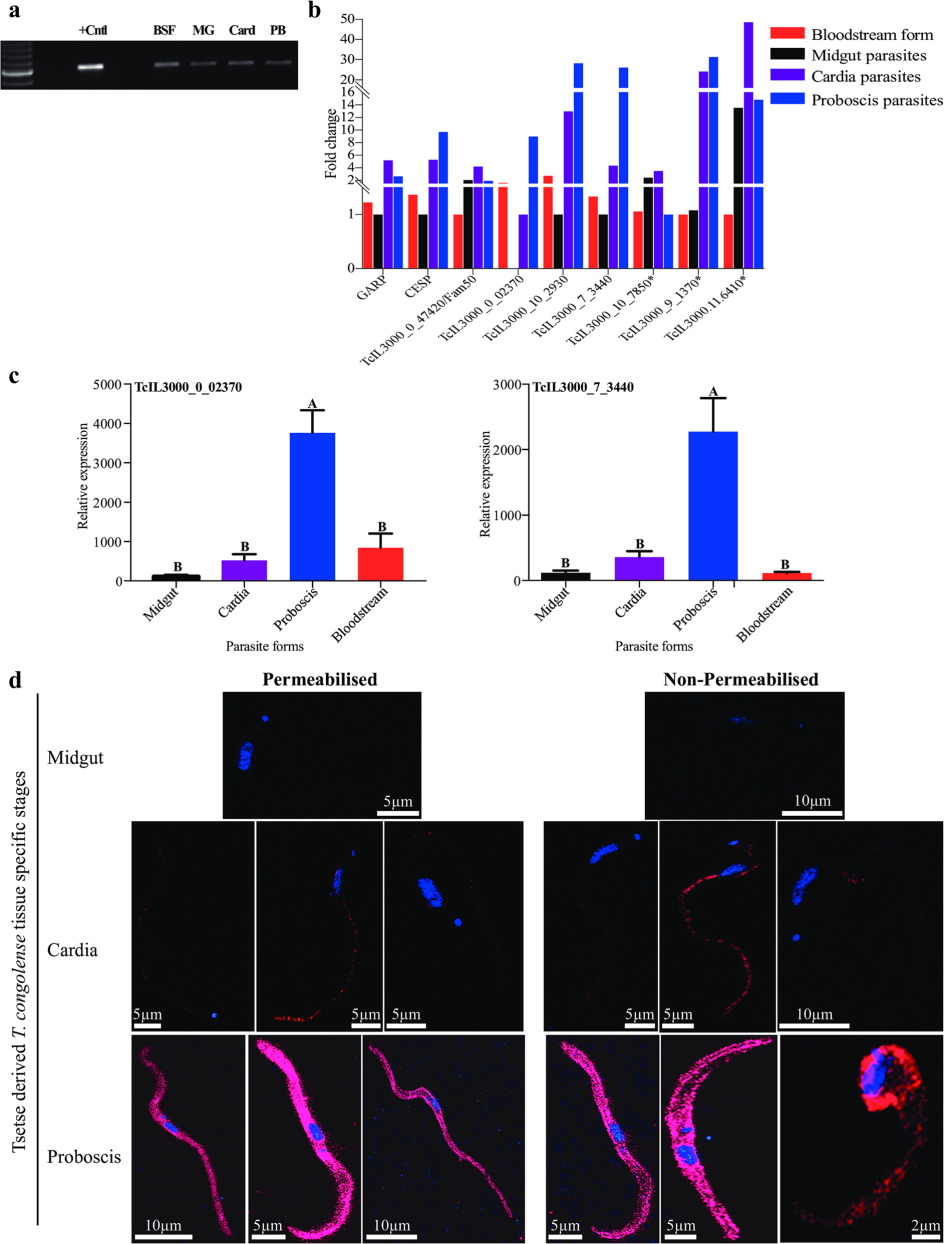
Expression analysis of selected CSP genes. **a** Expression levels of *T. congolense gapdh* from infected tsetse’s midgut, cardia and proboscis organs as well as bloodstream form parasites purified from infected mice blood. *Abbreviations*: BSF, bloodstream form; MG, tsetse midgut parasites; card, cardia parasites; PB, proboscis parasites. **b** Stage-regulated gene expression profiles for genes that putatively encode GPI-anchored and transmembrane proteins, normalized to *gapdh* levels as shown in A. * indicates transmembrane protein encoding genes. **c** Expression analysis of two GPI-anchored protein encoding genes *via* RT-qPCR. **d** Localization of *T. congolense* TcIL3000_0_02370 protein in parasites residing in the midgut, cardia and proboscis organs examined by immunofluorescent staining and confocal laser microscopy. Red indicates immunofluorescence staining of TcIL3000_0_02370, and blue indicates the DAPI staining of nucleus and kinetoplast

Semi-quantitative RT-PCR analysis was used to track the tissue and developmental stage-regulated expression of selected CSP encoding genes following normalization of samples using *T. congolense gapdh* across all tsetse tissues (midgut, cardia and proboscis) and BSF (Fig. 5a). Genes encoding GPI-anchor GARP, putative cell surface protein (TcIL3000_11_47420), and TM Receptor adenylate cyclase (GRESAG4, TcIL3000.11.6410) were most abundant in the cardia relative to other tsetse tissues and BSF parasites. Similarly, expression of TM Pteridine transporter (TcIL3000_10_7850) was higher in parasites colonizing the cardia and midgut than in the PB and BSF. The GPI-anchored Haptoglobin-hemoglobin (TcIL3000_10_2930), CESP family and hypothetical protein genes (TcIL3000_0_02370 and TcIL3000_7_3440) showed higher expression in PB-parasites than those colonizing other tissues (Fig. 5b).

The products of the two hypothetical genes (TcIL3000_0_02370 and TcIL3000_7_3440) have GPI-anchor motifs, and also showed higher expression in PB-parasites relative to C-parasites in both semi-quantitative RT-PCR and RNA-seq analysis. Hence the expression profiles of these genes were further tested in different infected fly tissues and BSF parasites by RT-qPCR. The ANOVA analysis of RT-qPCR results of these two genes indicate that they were both significantly (*F*_(3, 28)_ = 21.39, *P* < 0.0001) highly expressed by PB-parasites relative to parasites rest of the tsetse tissues and BSF (Fig. 5c).

We next expressed the TcIL3000_0_02370 protein in bacteria and generated rabbit polyclonal antibodies against the recombinant protein. These antibodies were then used to localize TcIL3000_0_02370 protein in parasites derived from distinct tsetse tissues. The hypothetical protein (TcIL3000_0_02370) which is predicted to be glycosylated, contain 453-amino acids and encoded a putative GPI-anchor (position 429) and signal peptide motif (positions 1–22). This putative protein was chosen for IFA analysis because its orthologue in *T. brucei* is expressed preferentially by metacyclic stage parasites [52]. Based on immunofluorescent and confocal laser microscopy analysis, the expression of TcIL3000_0_02370 protein was found to be localized on the surface of parasites residing in the PB, with minor staining also observed in C-parasites (Fig. 5d). The minor staining in the C-parasites may indicate that the expression of this protein probably starts in the population of parasites residing in the cardia and reaches its peak expression when parasites differentiate to EMF in the PB, as previously described in proteomic analysis of *T. congolense* four life-cycle stages [26].

## Discussion

In this study, we used high throughput RNA expression analysis to examine molecular differences of *T. congolense* developmental forms colonizing the cardia (C-parasites) and proboscis (PB-parasites) of *G. m. morsitans.* Our results reveal that C-parasites express genes that encode products linked with nucleotides/nucleosides, cell signaling and quorum sensing (QS), and several transport systems, suggesting parasite adaptation to varying nutritional environments in the vector. PB-parasites, on the other hand, express putative proteins associated with cell proliferation, in line with previous finding [12]. Greater cell division processes observed in PB-parasites may either enable multiplication of EMF or generation of short MCFs of *T. congolense* from long EMF [12]. In cultures of long attached EMFs, MCFs only appeared if short, attached trypomastigotes were present [67].

As extracellular parasites, African trypanosomes, including *T. congolense,* sense and respond appropriately to changes in their host environment. This feature is critical for the parasite to monitor nutrient availability, space and prevent accumulation of toxic metabolic waste for survival [68]. This process likely involves a repertoire of receptors, reporter molecule(s) and signaling pathways, with some evidence that cAMP and QS may also play important roles in this cascade [68, 69]. Increased expression of receptor-like adenylate cyclases (ACs; ESAG4/GRESSAG), QS and signal transduction proteins in C-parasites suggest that these individuals can also monitor their density [68] and engage in social behaviors as described for procyclic *T. brucei* [70–72]. The African trypanosome genomes encode an unusually expanded repertoire of ACs, with *T. brucei* encoding over 80 and *T. congolense* 45 [73]. This expansion only exists in the extracellular trypanosomes, but not in related intracellular kinetoplastids like *T. cruzi* and *Leishmania* spp. In *T. brucei,* one well studied member of subfamily of ACs is an expression site associated gene (ESAG4), which is a BSF stage specific flagellar pocket protein involved in disrupting host innate immunity [73]. Insect stage-specific *T. brucei* ACs have also been identified, suggesting similar roles during parasite development in the tsetse vector [13, 74]. ACs can also control cell division [75] and signal for social motility [76]. As such, upregulation of *T. congolense* ACs in the cardia may be linked to increased function of cAMP mediated signaling pathways and social motility [69], as C-parasites do not divide [12]. Aspects of QS and signal transduction-like social activities, including social motility, can facilitate trypanosome movement in the tsetse vector [70], and has been postulated for *T. brucei* procyclics in tsetse midgut [77, 78]. QS may also boost host nutrient accessibility while overcoming host defenses for survival [72]. Our results suggest that *T. congolense* C-parasites may also engage in social behaviors that can enable them to move from the cardia *en masse* to colonize the foregut as well as maximize the ability of these parasites to acquire nutrients from the cardia. The nutrient transporter proteins expressed by C-parasites may further enhance parasite uptake of metabolites in the cardia lumen.

We also detected expression of genes whose products potentially regulate parasite differentiation, e.g. RBPs, PADs and LPPs [59–61, 63, 79]. In *T. brucei*, overexpression of the gene encoding RNA Binding protein 6 (RBP6) using pLEW100 vector in in vitro cultured noninfectious PCF led to the generation of mammalian infective MCF [63]. The zinc finger protein, ZC3H20, regulates differentiation and growth of PCF [79], while ZC3H11 stabilizes heat-shock protein 70 and enables survival of BSF in the animal host [80]. PADs have been identified in *T. brucei*, where PAD2 enables differentiation of BSF cells to procyclics during the early stages of fly midgut infection [61]. The expression of all LPPs were induced in *T. congolense* PB-parasites similar to their *T. brucei* homologs, which are also induced in tsetse’s SG [60]. The increased expression of LPPs might be important for the uptake of phospholipids from PB by the attached parasites, in order to support parasite growth, proliferation and differentiation in the PB [81, 82].

Several African trypanosome CSPs that may be involved in host-parasite interactions have been identified [42]. Our analysis revealed that all members of Fam50 subfamily ‘iii’, ‘i’ and ‘GARP’ are induced in C-parasites while three members of the ‘CESP’ subfamily are induced in PB-parasites. This result contradicts previously held knowledge based on in vitro cultured *T. congolense* stages where GARP is preferentially expressed by EMF stage parasites [26, 83] in tsetse’s PB [12]. While the roles of GARP and ‘iii’ subfamily of proteins are unknown, CESP protein is thought to enable EMF attachment to the PB wall [84]. The levels of four invariant surface glycoproteins (ISGs), the Haptoglobin-hemoglobin receptor (HpHbR) and several VSGs, including three previously identified metacyclic specific-VSGs, are induced in PB-parasites [25, 26]. The VSGs may be a preadaptation strategy of the parasite for transmission to the animal host and evasion of host immunity [85]. The role of ISGs in *T. congolense* is unknown, but their *T. bruce*i homolog (ISG75) functions in suramin metabolism in BSFs [86]. The HpHbR of *T. congolense* has high affinity for hemoglobin and is strategically expressed on the surface of EMF parasites [26, 87], which appear in the PB and cibarium. In the PB, the HpHbR may enable the parasites to acquire heme from incoming blood during tsetse feeding [87, 88]. In *T. brucei,* HpHbR is BSF-specific, where it helps the parasite to acquire haptoglobin-hemoglobin complexes for heme [89]. In addition, several hypothetical CSPs were also induced in PB-parasites, notably TcIL3000_0_02370, which is thought to be involved in EMF to MCF development [26]. This single copy gene (TcIL3000_0_02370), has multi-copy orthologues (Tb927.7.360) in *T. brucei*’s genome and is preferentially expressed by SG colonizing parasites [52].

Given the small number of MCF parasites [90] that get deposited with saliva into the mammalian host bite site, the metacyclic developmental stage presents a bottleneck for transmission. If MCF-specific proteins can be effectively targeted at the bite site, it could block parasite infection establishment in animal hosts. Towards this end, studies in mice immunized with radiation-attenuated trypanosomes collected from animal blood 5-days post-challenge with infected tsetse showed protection against a subsequent parasite challenge. Again, mice that were challenged twice with *T. congolense* infected tsetse then treated with trypanocidal drugs after each challenge, and finally followed by homologous parasite challenge, resulted in sterile immunity [91, 92]. However, immunity to trypanosomes in these experiments was short-lived [93]. Such strategies have also been tried in *Plasmodium* spp*.* by targeting the CS antigen of sporozoites from mosquito SG [94, 95] and other antigens [96] to block transmission. Proteins expressed by immature parasite stages in the fly also present possible targets for genetic modification to prevent parasite maturation in the vector [97]. However, it should be noted that *T. congolense* populations, particularly those in tsetse’s PB, are variable, and gene expression analyses of these parasites is complex and difficult to tease apart.

## Conclusions

Collectively, our results provide insight into *T. congolense* gene expression profiles in two distinct niches within the tsetse vector. Results from C-parasites suggest that *T. congolense* parasites in the cardia may exhibit social motility. In addition, we identified a number of genes encoding putative cell surface proteins and proteins associated with parasite differentiations that may regulate *T. congolense* developmental processes in tsetse fly. Genes encoding transporter proteins abundantly expressed by C-parasites may aid the parasite in acquiring nutrients from the cardia. Our findings together with the previous proteome and transcriptomic data based on in vitro cultured parasites [25, 26], provide resources to facilitate further research into the life-cycle of *T. congolense* in the tsetse vector and expand the toolbox for transmission control.

## Additional files

Additional file 1: **Text S1.** List of primers used in this study. (DOCX 105 kb)

Additional file 2: **Text S2.** Validation of the transcriptome. (DOCX 127 kb)

Additional file 3: **Figure S1.** Overview of *T. congolense* transcriptome analysis from infected *G. morsitans* cardia or proboscis. **a** Processing of RNA-seq reads from *G. morsitans* cardia or proboscis 28 days post-infection with *T. congolense* TC13. **b** Mapping statistics of *T. congolense* TC13 reads from *G. morsitans* cardia or proboscis to the *T. congolense* transcripts. **c** Spatial distribution of the most abundantly (99 percentile RPKM) expressed *T. congolense* TC13 genes between cardia or proboscis infections. Shared genes with higher expression in cardia or proboscis are indicated with ‘*’ in respective directions. (TIF 1642 kb)

Additional file 4: **Table S1.** The top 99 percentile (RPKM) most highly expressed genes in cardia and proboscis. (XLSX 63 kb)

Additional file 5: **Table S2**. Differentially expressed genes between C- and PB-parasites. **Sheet 1.** Differently Expressed genes (FC ≥ 2) between C-parasites and PB-parasites with FDR, *P* < 0.05. **Sheet 2.** Moderately differently expressed genes (FC < 2) between C-parasites and PB-parasites with FDR, *P* < 0.05. **Sheet 3.** Differently expressed genes (FC ≥ 2) between C-parasites and PB-parasites with FDR, *P* < 0.05 only with CLC-Genomics tool. **Sheet 4.** Differently expressed genes (FC ≥ 2) between C-parasites and PB-parasites with FDR, *P* < 0.05 only with EdgaR software. **Sheet 5.** Differently expressed genes (FC ≥ 2) between C-parasites and PB-parasites with FDR, *P* < 0.05 with proteomics data from [25]. (XLSX 1187 kb)

Additional file 6: **Table S3.** Gene ontology analysis of differentially expressed gene products between C-parasites and PB-parasites. (XLSX 31 kb)

Additional file 7: **Table S4.** Analysis of genes coding for proteins associated with differentiation. (XLSX 62 kb)

Additional file 8: **Table S5.** Expression analysis of *T. congolense* genes encoding cell surface proteins. (XLSX 90 kb)

Additional file 9: **Figure S2.** Heatmaps showing expression of *T. congolense-*specific genes belonging to Cell Surface Phylome families 17–21. The genes in these families were obtained [42] deposited in GeneDB [98]. The expression profiles consist of respective Log_2_ transformed RPKM clustered using Euclidean distance calculation and ward.D clustering methods. (TIF 1937 kb)

## Abbreviations

AAT: Animal African trypanosomosis
AC: Receptor-like adenylate cyclase
BCA: Bicinchoninic acid assay
BSF: Bloodstream form trypanosomes
cDNA: Complemetary deoxyribonucleic acid
CESP: Congolense ecpimastigote specific protein
CSP: Cell surface protein
DAPI: 4',6-diamidino-2-phenylindole
DE: Differentially expressed genes
EMF: Epimastigote form trypanosome
FDR: False discovery rate
GARP: Glutamic acid alanine rich protein
GO: Gene Ontology
GPI: Glycosylphosphatidyl inositol
HpHbR: Haptoglobin-hemoglobin receptor
IPTG: Isopropyl-β-thiogalactoside
ISG: Invariant surface glycoprotein
KEGG: Kyoto Encyclopedia of Genes and Genomes
LPP: Lipid phosphate phosphatases
MCF: Metacyclic form trypanosomes
m-VSG: Metacyclic-specific variant surface glycoprotein
NCBI: Centre for Biotechnology Information
PAD: Proteins associated with differentiation
PB: Proboscis
PCF: Procyclic form trypanosome
PCR: Polymerase chain reaction
PSG: Phosphate buffered saline glucose
QS: Quorum sensing
RBP: RNA-binding protein
RPKM: Reads per kilobase of transcripts per million mapped reads
RT-qPCR: Real-time quantitation polymerase chain reaction
TM: Transmembrane
VAT: Variant antigenic type
VSG: Variant surface glycoprotein
