# Supplementary material for: Expression profiling of Trypanosoma congolense genes during development in the tsetse fly vector Glossina morsitans morsitans
Source: Parasit Vectors. 2018 Jul 3;11:380. doi: 10.1186/s13071-018-2964-8 (PMC6029126; doi:10.1186/s13071-018-2964-8)
Supplement: Supplementary file 1 — Text S1. List of primers used in this study. (DOCX 105 kb) [file 13071_2018_2964_MOESM1_ESM.docx]

**Text S1.**

Primer list used for *T. congolense* transcriptome validation, semi-quantitative RT-PCR and cloning experiments

| **Putative product name** | **TrytripDB gene ID** | **Forward primer** | **Reverse primer** |
| --- | --- | --- | --- |
| **For *Trypanosoma congolense* validation** | | | |
| 60S Ribosomal protein | TcIL3000_0_32580 | CTTCCGCACTGTGACAAACG | TAAATCATGCGGGCGAGGAG |
| GAPDH | TcIL3000_10_5910 | GATGCCAGAGCGGGTATCTC | TGCGAGATGAGGTCCAACAC |
| Hypothetical protein | TcIL3000_0_02370 | ACAGCAGCGTAACGTCTGAG | TTCGAAAACACCTCCACCGT |
| Hypothetical protein | TcIL3000_7_3440 | TTCGCTGGCACTGAATGACT | GCTAACAACATGCAGCTCCG |
| Hypothetical protein | TcIL3000_0_37480 | CACGGGCTTCCTGATCGTAT | AACCGCAACTAGGCACAACT |
| Leucine alanine rich protein | TcIL3000_7_2150 | TGCCAGCTCTCACTTTGTCA | TACTGTGCACACAAGGGGAC |
| RNA-binding protein | TcIL3000.11.14360 | CGCATCCGTATGTACCGTCA | CTGTCCTTCTCAAGGGAGCG |
| BTI protein | TcIL3000_0_56730 | GGGCTAAGCAAAGCCAACAC | GGAGCCAATGAGACCACCAA |
| Amino acid transporter | TcIL3000_10_13970 | GCAGTGGACTTGTGATGGGA | CTCGTGGCTTCCTCGAAACT |
| Cytochrome C oxidase | TcIL3000_1_1680 | GTTTGGCAATGAGGTGCGTT | GCAATCATCGCGTTCTCGAC |
| **For Semi-quantitative RT-PCR** | | | |
| GAPDH |  | TGCCGTGGTGGACATGAACA | ACCATACCAACAGCCTTTGC |
| Hypothetical protein | TcIL3000_0_02370 | AGTCGGAGAAAGCACGAAGG | TTCCTTCGCTCGCTGAACTT |
| GARP |  | CATGTCCCGTGTCCTACACC | TCCAGAGACTTCAGCCCCTT |
| CESP |  | TGTTGAAGCTGAGGTGACTGC | ACAGCACACCAACAAGTGCG |
| Hepatoglobulin-Haemoglobulin | TcIL3000_10_2930 | CCATCATGCATTGGAGCACG | GGCAACCGTCCCCATAAGAA |
| Pteridine transporter | TcIL3000_10_7850 | CCATCACAGAACCTACGGCA | ACAAGCGCGACAGAAGTACA |
| Hypothetical protein | TcIL3000_9_1370 | AAAGACCAGAGAAGGATGTGACCA | CACGAGGAAGGGCGCAAGA |
| Putative cell surface family | TcIL3000_0_47420/ family | AACCGTCTCGAATGCCTCTG | CATCGCTATCACTGGGCTCC |
| Gresag4 | TcIL3000.11.6410 | TGTTCTGCTTTTTGGCTCGC | GCGCGATACGCAACTTCATT |
| Hypothetical protein | TcIL3000_7_3440 | CTCATGCTTTTCGGCGGTTT | GTTGTATCCTCGCTCCCGTC |
| **Cloning primer** | | | |
| Hypothetical protein | TcIL3000_0_02370 | ATTCG**GGATCC**CGAGATGATGAGAACGAGAAG | CAC**CTCGAG**TGCATCATCAGAAGTATTTCCCCCTCC |
