# Supplementary material for: Expression profiling of Trypanosoma congolense genes during development in the tsetse fly vector Glossina morsitans morsitans
Source: Parasit Vectors. 2018 Jul 3;11:380. doi: 10.1186/s13071-018-2964-8 (PMC6029126; doi:10.1186/s13071-018-2964-8)
Supplement: Supplementary file 2 — Text S2. Validation of the transcriptome. (DOCX 127 kb) [file 13071_2018_2964_MOESM2_ESM.docx]

**Text S2**

Validation of the *Trypanosoma congolense* RNA-seq results with qPCR. The expression values (log_2_ ratios) for eight genes are plotted against qPCR values (log_2_ ratios). The Pearson correlation coefficient (**R=0.991496855**) and Goodness fit, **R^2^ = 0.98307** obtained are quite high indicating high correlation. These results indicate that the qPCR correctly validated the T. congolense RNA-seq data of parasites from the cardia and probosces.

| **Putative product name** | **TrytripDB gene ID** | **Fold change (RT-qPCR)** | **Log2 RT-qPCR** | **Fold Change (RNA-seq)** | **Log_2_ RNA-seq)** | **EdgaR (Log Fold change)** |
| --- | --- | --- | --- | --- | --- | --- |
| Hypothetical protein | TcIL3000_0_02370 | -10.42625 | -3.38215 | -10.53442 | -3.39704 | -3.35077 |
| Hypothetical protein | TcIL3000_7_3440 | -8.31164 | -3.05513 | -8.41561 | -3.07307 | -3.00569 |
| Hypothetical protein | TcIL3000_0_37480 | -5.13985 | -2.36173 | -5.54540 | -2.47129 | -2.39750 |
| Leucin alanine rich protein | TcIL3000_7_2150 | -3.89389 | -1.96121 | -5.87844 | -2.55543 | -2.48382 |
| RNA-binding protein | TcIL3000.11.14360 | -1.43243 | -0.51847 | -1.64684 | -0.71970 | -0.64729 |
| BT1 family – Pteridine transportor | TcIL3000_0_56730 | 2.30594 | 1.20535 | 2.83387 | 1.50277 | 1.57126 |
| Cytochrome C oxidase | TcIL3000_1_1680 | 1.70600 | 0.77062 | 2.20582 | 1.14131 | 1.22392 |
| Amino acid transporter | TcIL3000_10_13970 | 1.37651 | 0.46101 | 1.80276 | 0.85021 | 0.92714 |
