# Supplementary figures and images for: Expression profiling of Trypanosoma congolense genes during development in the tsetse fly vector Glossina morsitans morsitans
Source: Parasit Vectors. 2018 Jul 3;11:380. doi: 10.1186/s13071-018-2964-8 (PMC6029126; doi:10.1186/s13071-018-2964-8)

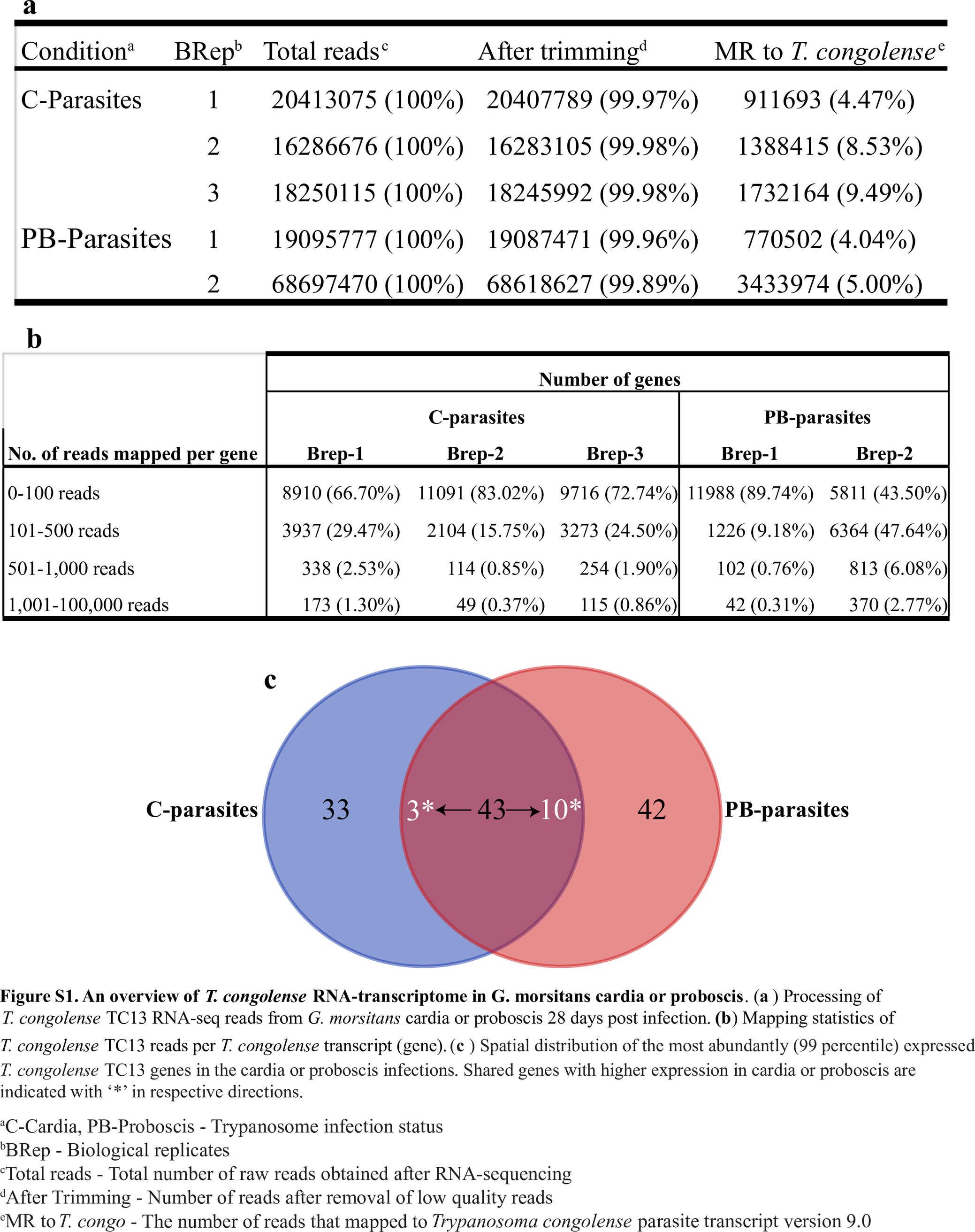

Supplement: Supplementary file 3 — Figure S1. Overview of T. congolense transcriptome analysis from infected G. morsitans cardia or proboscis. a Processing of RNA-seq reads from G. morsitans cardia or proboscis 28 days post-infection with T. congolense TC13. b Mapping statistics of T. congolense TC13 reads from G. morsitans cardia or proboscis to the T. congolense transcripts. c Spatial distribution of the most abundantly (99 percentile RPKM) expressed T. congolense TC13 genes between cardia or proboscis infections. Shared genes with higher expression in cardia or proboscis are indicated with ‘*’ in respective directions. (TIF 1642 kb) [file 13071_2018_2964_MOESM3_ESM.tif]

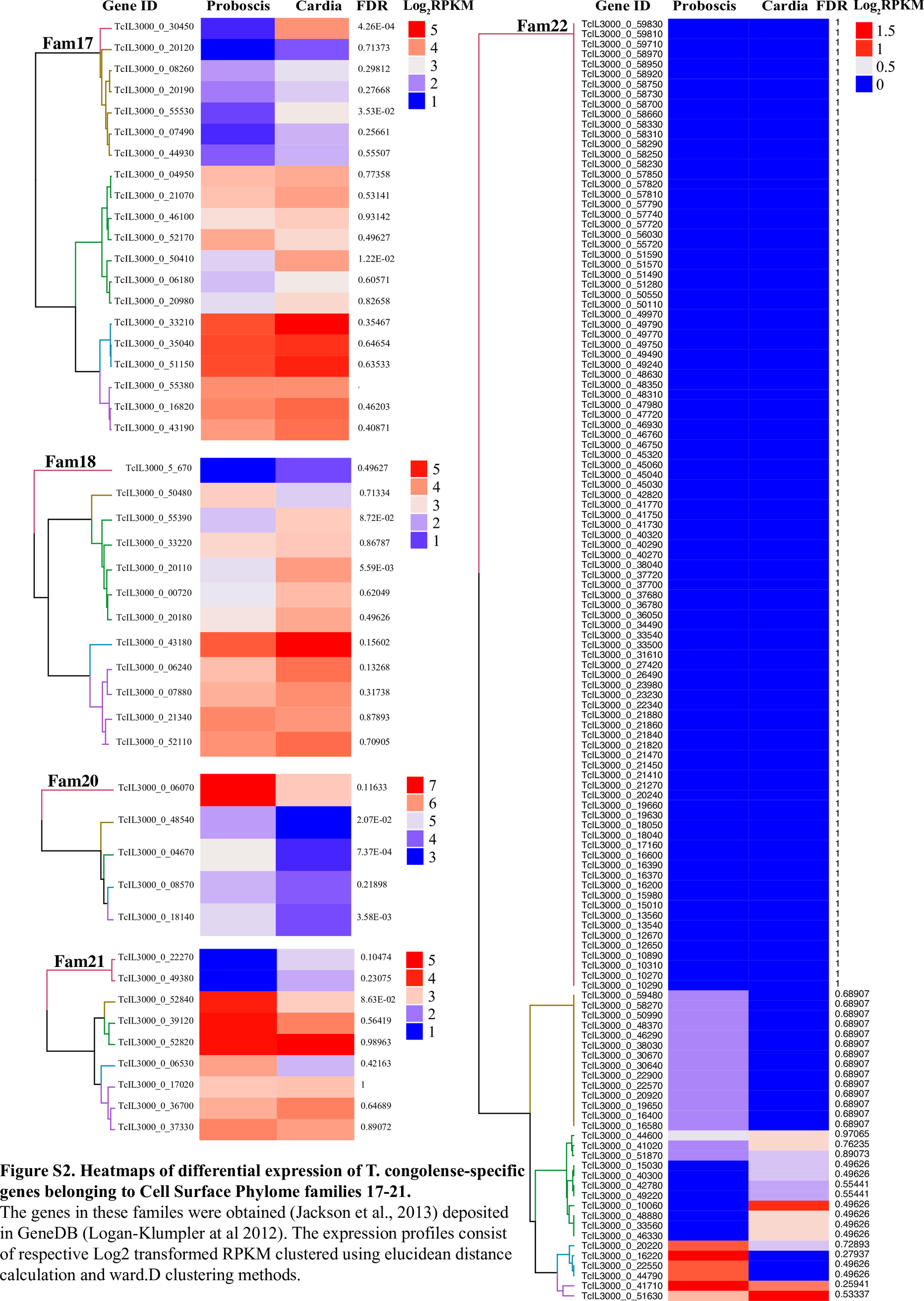

Supplement: Supplementary file 9 — Figure S2. Heatmaps showing expression of T. congolense-specific genes belonging to Cell Surface Phylome families 17–21. The genes in these families were obtained [42] deposited in GeneDB [98]. The expression profiles consist of respective Log2 transformed RPKM clustered using Euclidean distance calculation and ward.D clustering methods. (TIF 1937 kb) [file 13071_2018_2964_MOESM9_ESM.tif]
